# Supplementary material for: Additive GaN Solid Immersion Lenses for Enhanced Photon Extraction Efficiency from Diamond Color Centers
Source: ACS Photonics. 2023 Aug 30;10(9):3374–83. doi: 10.1021/acsphotonics.3c00854 (PMC10515637; doi:10.1021/acsphotonics.3c00854)
Supplement: Supplementary file 1 — ph3c00854_si_001.pdf [file ph3c00854_si_001.pdf]

Supporting Information: Additive GaN solid immersion lenses for enhanced photon extraction efficiency from diamond color centers

Xingrui Cheng,[†,‡,⊥] Nils Kolja Wessling,[¶,⊥] Saptarsi Ghosh,[§] Andrew R. Kirkpatrick,[†,‡] Menno J. Kappers,[§] Yashna N. D. Lekhai,[‖] Gavin W. Morley,[‖] Rachel A. Oliver,[§] Jason M. Smith,[‡] Martin D. Dawson,[¶] Patrick S. Salter,[†] and Michael J. Strain[*,¶]

†*Department of Engineering Science, University of Oxford, Oxford OX1 3PH, UK*

‡*Department of Materials, University of Oxford, Oxford OX1 3PJ, UK*

¶*Institute of Photonics, Department of Physics, University of Strathclyde, Glasgow G1 1RD, UK*

§*Cambridge centre for Gallium Nitride, University of Cambridge, Cambridge CB3 0FS, UK*

‖*Department of Physics, University of Warwick, Coventry CV4 7AL, UK*

⊥*Both authors contributed equally to this work*

E-mail: michael.strain@strath.ac.uk

Abstract

This document provides further details on the experimental setups, an analysis of displacement and different lens geometries with FDTD simulations, additional AFM

and PL data on the discussed emitters and PL spectra taken on GaN/AlGaN/AlN thin films at room temperature.

Confocal setups used for photoluminescence measurements

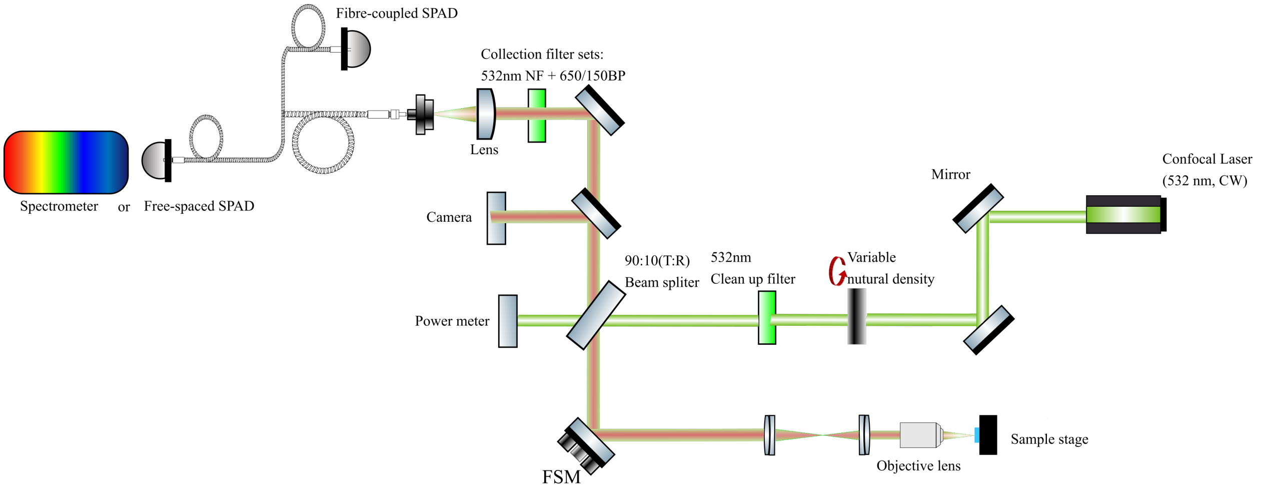

Fig. S1: Home-built confocal microscope for dual air (NA = 0.95) and oil (NA = 1.25) objective lens

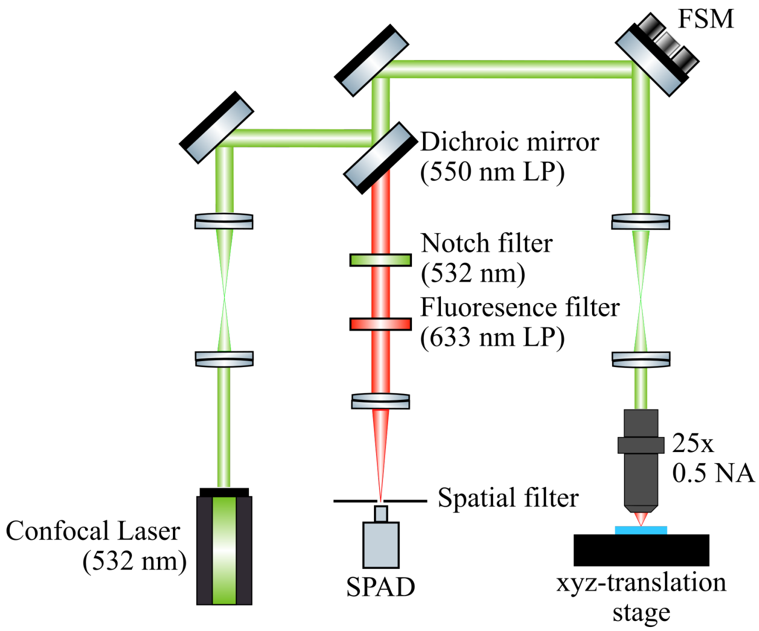

Fig. S2: Home-built confocal microscope for NA = 0.5

The confocal photoluminescence setup that was used to gather the power series on the two doublet emitters in addition to the presented autocorrelation and spectral measurements with a NA = 0.95 air lens is depicted in Fig. S1. NA = 1.25 oil lens were used in the same setup to get spatial distribution information of the emitter of interest with best signal to noise ratio. Here, a continous wave 532 nm laser (GEM532) excitation source with 1 mW output power was used, the fluorescence signal was collected back through the same objectives lens and spectral filtering of the excitation laser is applied before detection.
Fig. S2 shows the setup used for measurements with the NA = 0.5 air objective. A continuous-wave 532 nm laser (GEM532) with 6 mW output power was used as excitation source. Photoluminescence maps are taken with both setups.

Displacement of emitter with respect to the mid point of the lens sphere and the influence of an air gap on the collection efficiency

In this section we present additional FDTD simulations to investigate the effects of small displacements of two dipole emitters mimicking a NV^- centre in a diamond crystal with (100) surface orientation below a GaN SIL which radius of curvature is matched to the emitter depth of 5 μm. Two separate simulations are run and averaged to account for the two different dipole moments of the NV^- center, one of which is tilted by (90-54.7) ° with respect to the surface normal (the NV axis is tilted by 54.7 °, but both dipole moments are orientated perpendicular to the NV axis). Cross sections and far field projection are taken at $\lambda = 700$ nm wavelength. The expected collection efficiency is calculated by averaging the transmission through the detector surface above the lens (indicated in green) multiplied with the projected far field distribution from the same detector, choosing $\lambda = 650 - 750$ nm wavelength to match the spectral emission region of the NV^- centre. Both transmission and

far field projection are found to be insensitive towards wavelength changes in this spectral regime, allowing us to ignore the spectral density distribution of the emission spectrum. Lateral displacement is discussed in Fig. S3, vertical displacement in Fig. S4. We illustrate the effects of a slightly larger radius of curvature accompanied by a larger diameter of the micro-lens on the collection improvement as function of an air gap between diamond and the AlN bottom surface of the lens platelet in Fig. S5.

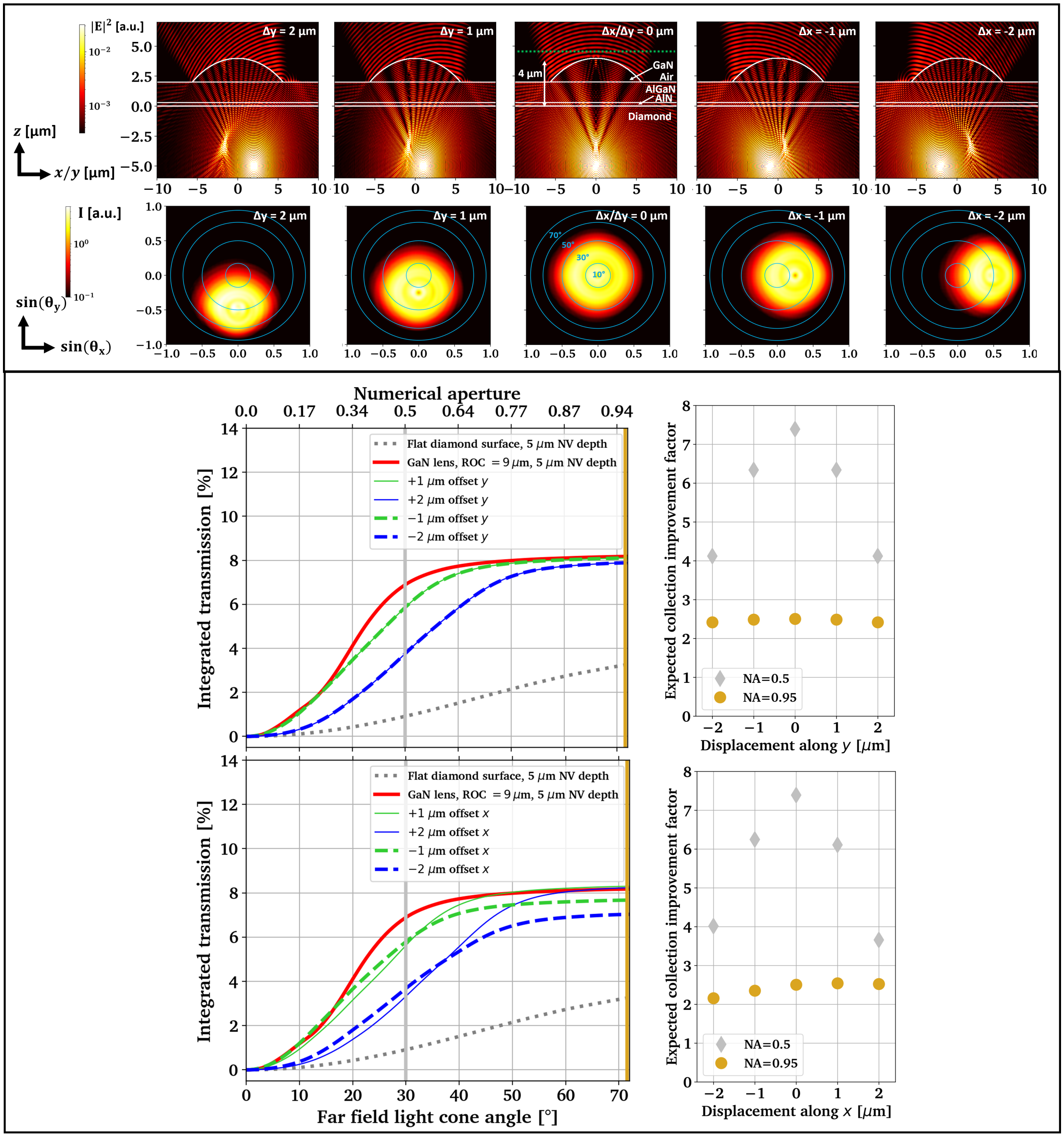

Fig. S3: Influence of x and y displacement on the expected collection efficiency from the two dipole emitter. The radius of curvature of the GaN micro-lens is matched to the emitter depth of $5\,\mu$m. Cross sections are taken at $\lambda = 700\,$nm wavelength and the transmission is averaged between $\lambda = 650 - 750\,$nm wavelength.

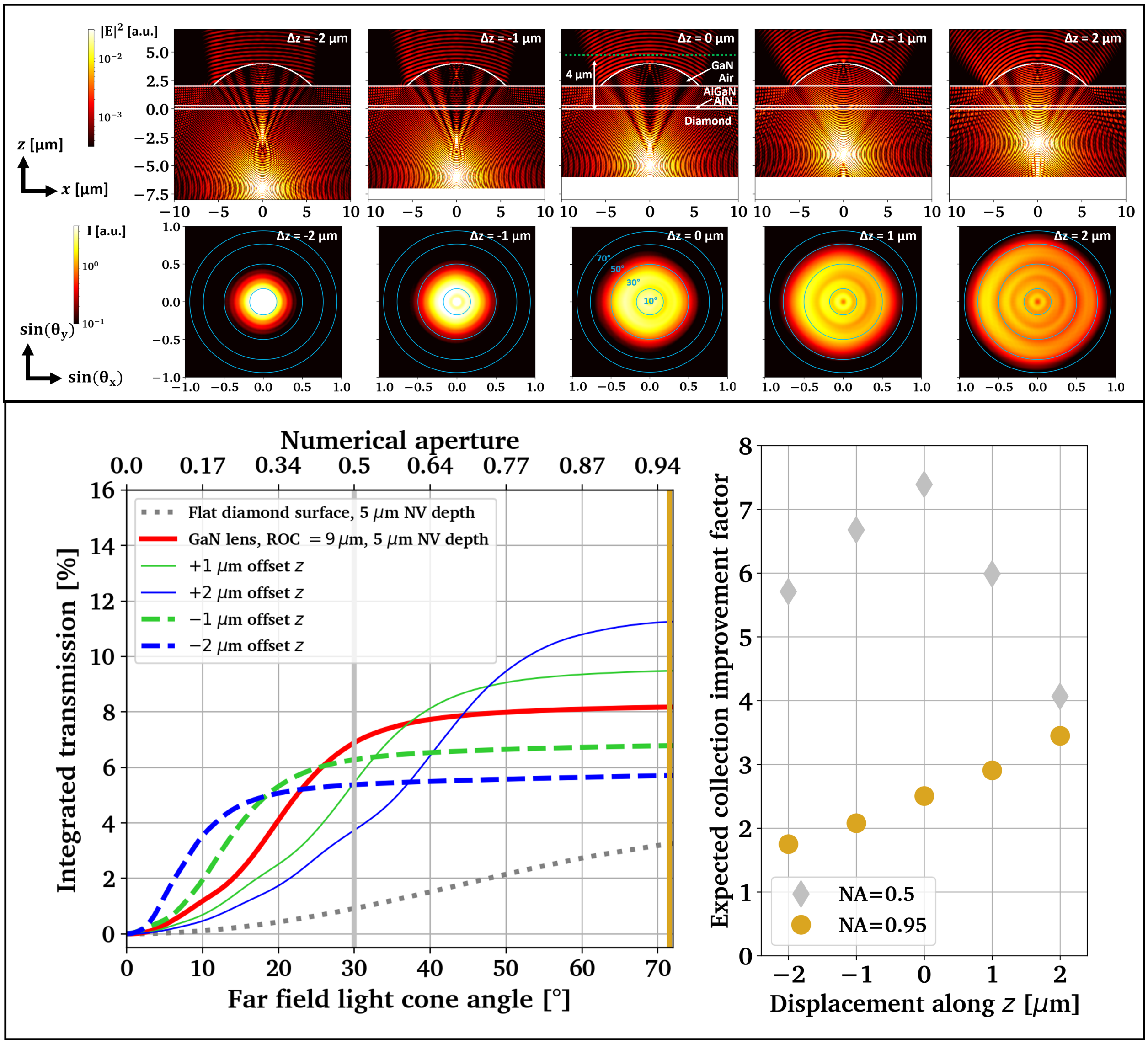

Fig. S4: Influence of z displacement on the expected collection efficiency from the two dipole emitter. The radius of curvature of the GaN micro-lens is matched to the emitter depth of $5\,\mu$m. Cross sections are taken at $\lambda = 700\,$nm wavelength and the transmission is averaged between $\lambda = 650 - 750\,$nm wavelength.

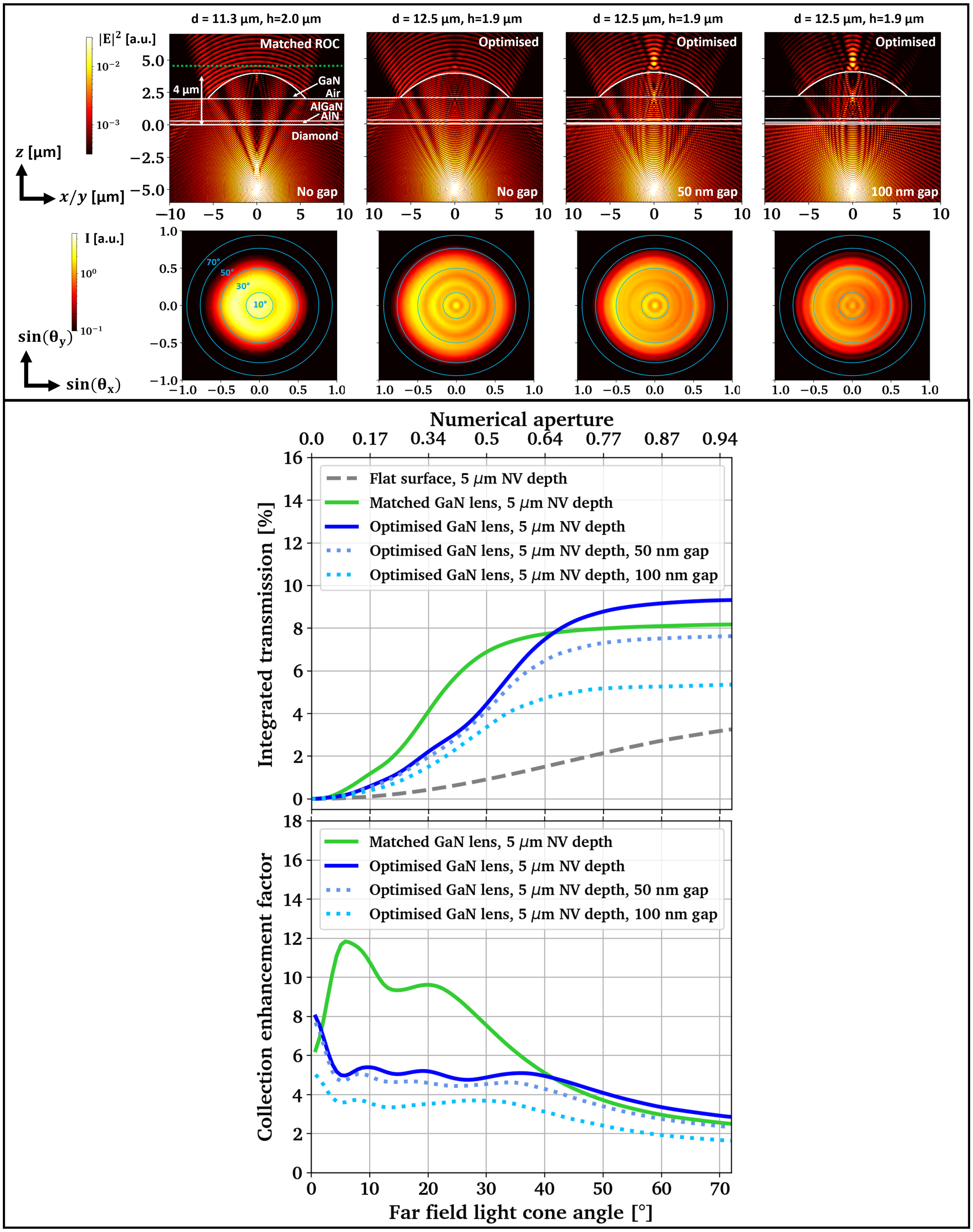

Fig. S5: FDTD simulations taking the measured AFM profile from the lens above emitter pair 1 (Fig. S6,a and Fig. 4 in the main text) into account. The most left cross section and far field plot show the previously discussed GaN lens with matched radius of curvature for reference. Additionally, the effect of an air gap between diamond and AlN bottom layer of the GaN lens layer stack is investigated on the right hand side of the figure. Cross sections are taken at $\lambda = 700\,\text{nm}$ wavelength and the transmission is averaged between $\lambda = 650 - 750\,\text{nm}$ wavelength.

Additional photoluminescence and AFM measurements of SILs and emitters

To provide some additional data from the investigated emitters, Fig. S6 a) includes a larger field of view of the photo luminescence plots shown in Fig. 4 a) in the main text as well as an AFM line scan of this slightly larger SIL. Fig. S6 b) includes photoluminescence maps and an AFM line scan of the second doublet emitter discussed in less detail in the main text, while Fig. S6 c) illustrates the NA dependency of the PL map taken on the graphitised emitter spot discussed in Fig. 3 in the main text.

Room temperature spectral measurements of transfer printed GaN lenses and AlGaN/AlN membranes on single crystalline diamond are shown in Fig. S7. A 532 nm CW excitation laser is used with a 550 nm long pass filter and 532 nm notch filter in a confocal arrangement.

Fig. S8 a) and b) show the HBT measurements before and after lens integration, respectively. Before integration we find $g^2(0) = 0.72$ at high pump power, with the high pump power leading to significant charge state conversion and to the introduction of additional background light, both contributing to reduced visibility of the $g^2(0)$ value.

Due to the large depth of $> 5\,\mu$m of the emitter below the SIL we dont expect significant PL background contributions from the micro-lens after integration if high NA objective lenses are used. We can confirm this by comparing the PL data collected with the oil immersion objective (NA = 1.25) without SIL and the data collected after lens integration with an air objective (NA = 0.95) where we find very similar SNR.

After lens integration (Fig. S8 b) we find an asymmetric spike-like feature on the right-hand side of the histogram at $\tau \approx 20$ ns, which is most probably due to optical cross-talk between the SPADs. The break down flash in one SPAD is emitting IR photons which can be detected by the second SPAD. The asymmetry between the left and right hand side of the histogram arises from unbalanced reflections and coupling between the two different SPADs used: one

is free space coupled and the other fibre coupled. Similar spiking features can be observed in the histogram in Fig. S8 a) before lens integration at $\tau \approx \pm 20\,\text{ns}$, indicating that this effect is not due to the GaN SIL.

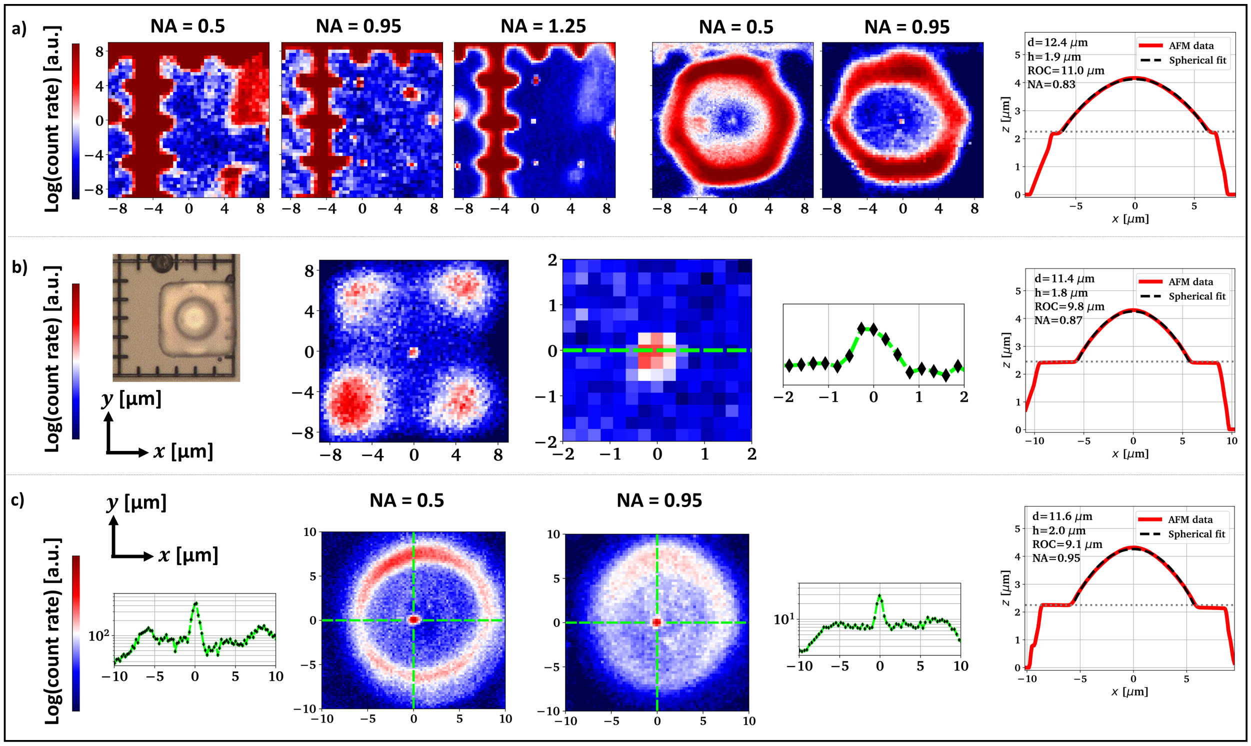

Fig. S6: Photoluminescence and AFM measurements regarding a) doublet emitter 1 shown in Fig. 4, b) doublet emitter 2 included in the power saturation measurement shown in Fig. 5 a), c) the micro-lens in the top left quadrant discussed in Fig. 3.

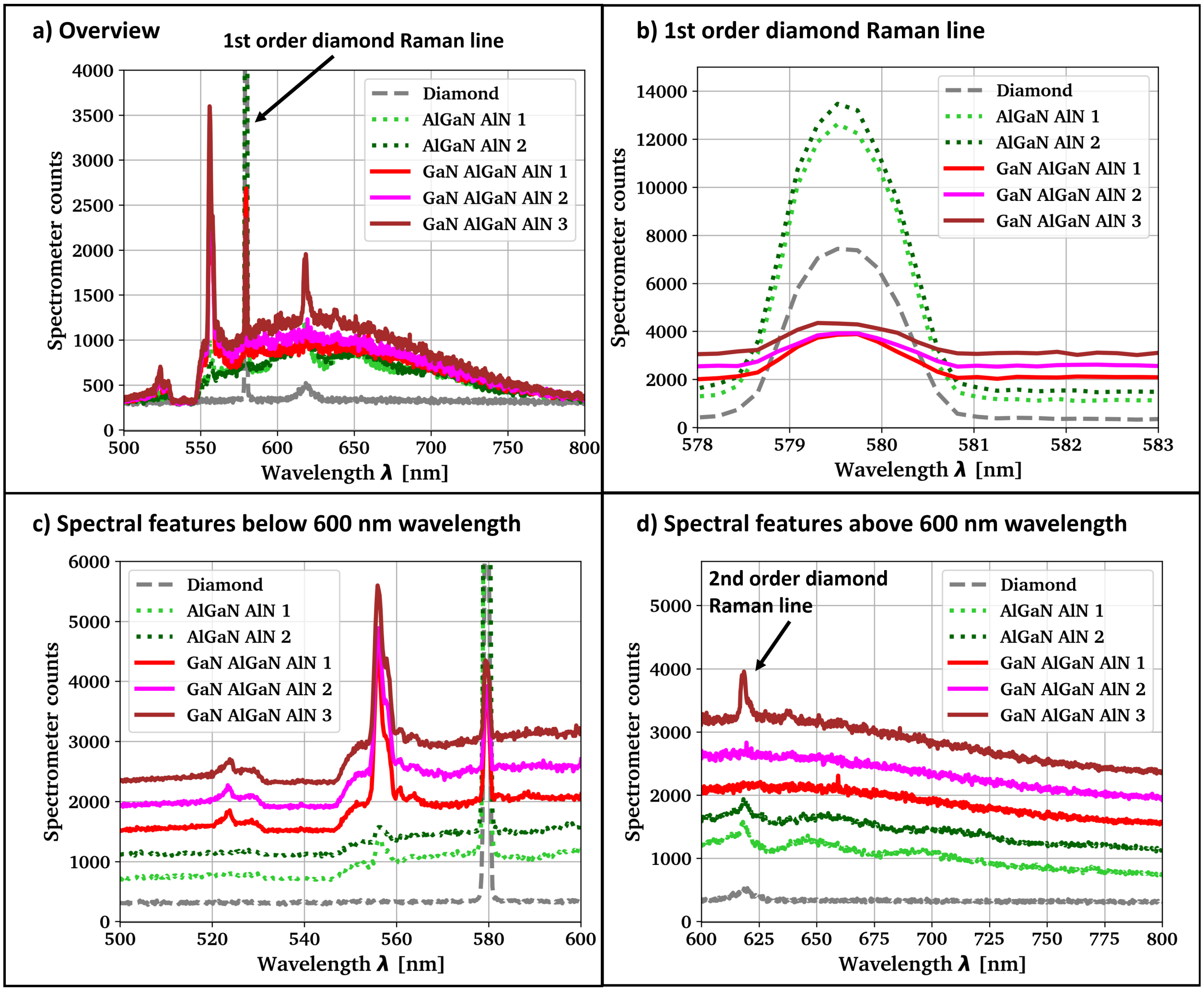

Fig. S7: Room temperature photoluminescence spectra of transferprinted GaN lenses on AlGaN/AlN mesa structures on a single crystalline CVD grown polished diamond sample. a)-d) show the same spectra, which are separated linearly in b)-d) for better visibility.

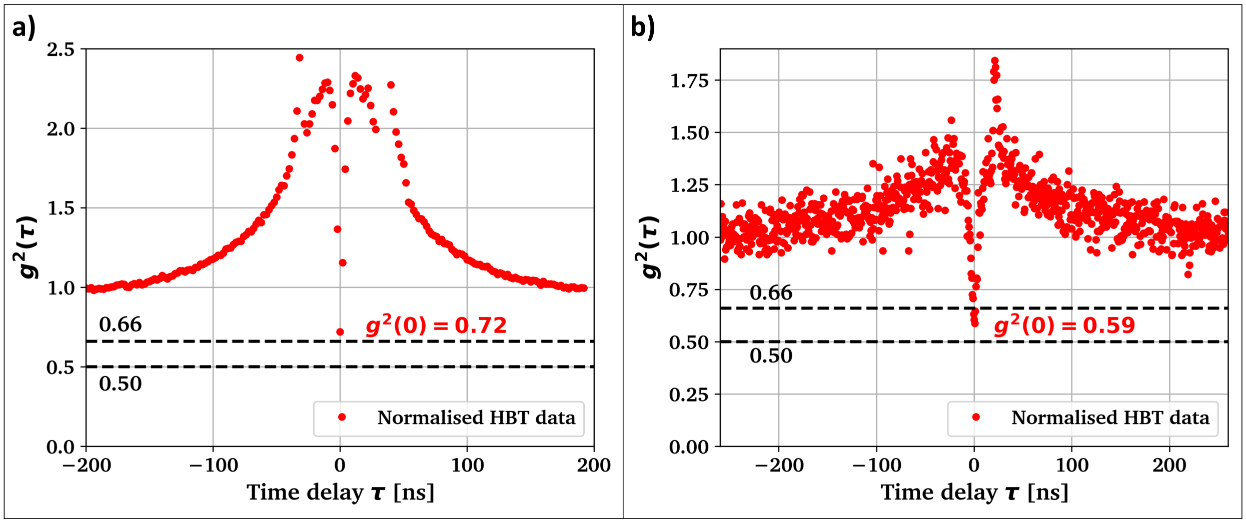

Fig. S8: Room temperature autocorrelation measurements on the same doublet emitter a) before and b) after GaN SIL integration using the objective with NA = 0.95. The histogram shown in b) is the corresponding raw data to the autocorrelation measurement shown in Fig. 4 b) in the main text.

Detailed FDTD simulation results for emitters at different depth and increased GaN epilayer thickness

To provide a more detailed overview of the simulation results for dipole emitters in different depth below the diamond surface and the potential arising from a thicker GaN epilayer, we summarised the absolute collection efficiency and collection enhancement as function of the NA of the collection optics for both (100) and (111) crystal direction in Fig. S9. The bottom left plot is already shown in the main text in Fig. 5 c).

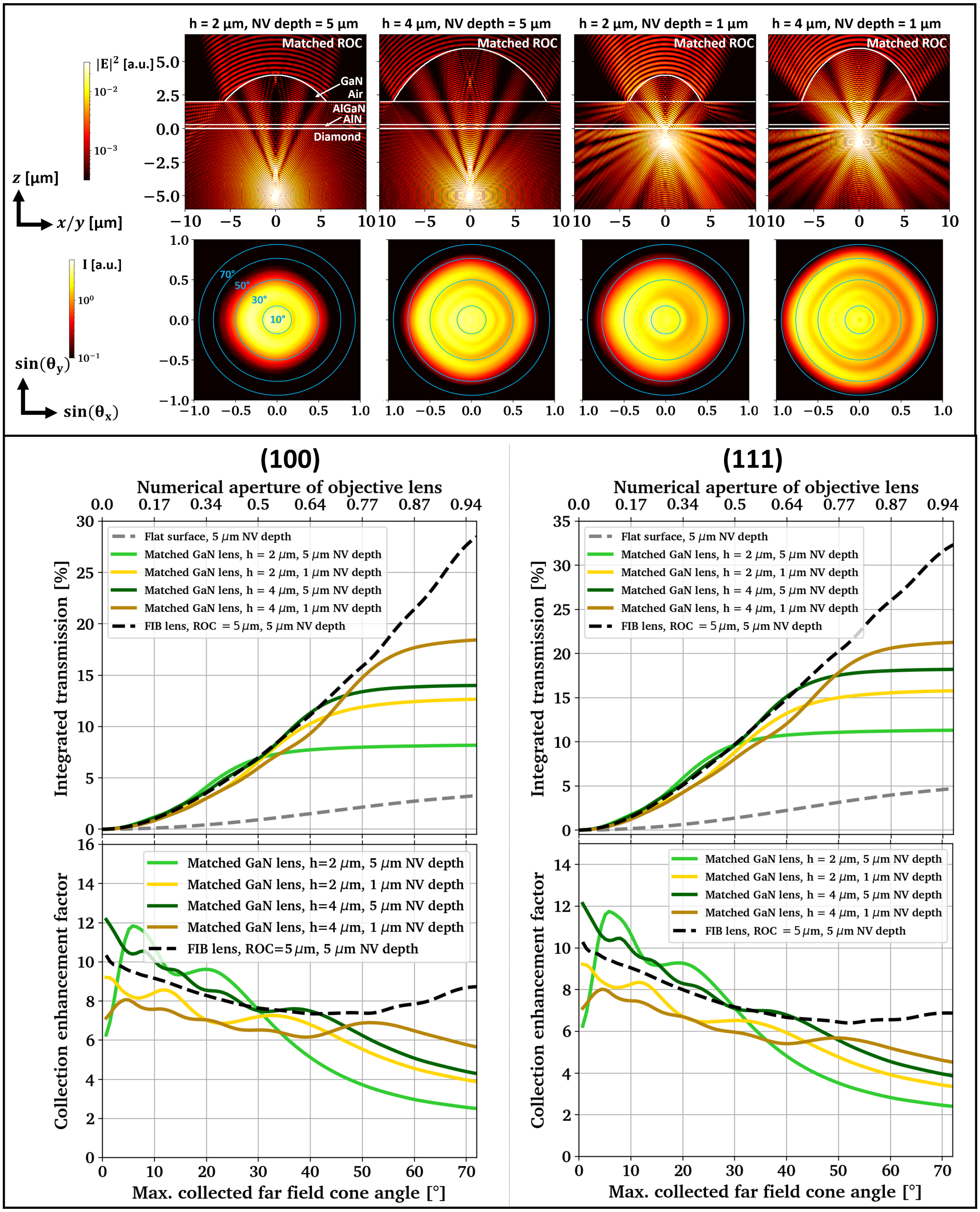

Fig. S9: Light collection and its enhancement in dependence of emitter depth and GaN lens height expected form FDTD simulations. The emitter position is matched to the geometric center of each lens. The cross sections are taken for (100) diamond surface orientation, while the bottom plots distinguish between (100) and (111), assuming an ideally aligned emitter in the (111) case. Cross sections are taken at $\lambda = 700\,\text{nm}$ wavelength and the transmission is averaged between $\lambda = 650 - 750\,\text{nm}$ wavelength.
